# Comprehensive evaluation of physiological traits under nitrogen stress and participation of linolenic acid in nitrogen-deficiency response in wheat seedlings

**DOI:** 10.1186/s12870-020-02717-5

**Published:** 2020-11-03

**Authors:** Xiaoxiao Liu, Shiwen Wang, Xiping Deng, Zhiyong Zhang, Lina Yin

**Affiliations:** 1 State Key Laboratory of Soil Erosion and Dryland Farming on the Loess Plateau, Institute of Soil and Water Conservation, Northwest A&F University, Yangling, 712100 Shaanxi China; 2 University of the Chinese Academy of Sciences, Beijing, 100049 China; 3 Institute of Soil and Water Conservation, Chinese Academy of Sciences and Ministry of Water Resources, Yangling, 712100 Shaanxi China; 4 Henan Key Laboratory for Molecular Ecology and Germplasm Innovation of cotton and wheat, Henan Collaborative Innovation Center of Modern Biological Breeding, Henan Institute of Science and Technology, Xinxiang, 453003 Henan China

**Keywords:** Wheat, Nitrogen deficiency, Nitrogen-deficient tolerance, Linolenic acid, Principal component analysis

## Abstract

**Background:**

Nitrogen (N) deficiency is a major constraint for plant production in many areas. Developing the new crop genotypes with high productivity under N deficiency is an important approach to maintain agricultural production. Therefore, understanding how plant response to N deficiency and the mechanism of N-deficiency tolerance are very important for sustainable development of modern crop production.

**Results:**

In this study, the physiological responses and fatty acid composition were investigated in 24 wheat cultivars under N-deficient stress. Through Pearson’s correlation analysis and principal component analysis, the responses of 24 wheat cultivars were evaluated. The results showed that the plant growth and carbohydrate metabolism were all differently affected by N deficiency in all tested wheat cultivars. The seedlings that had high shoot biomass also maintained high level of chlorophyll content under N deficiency. Moreover, the changes in fatty acid composition, especially the linolenic acid (18:3) and the double bond index (DBI), showed close positive correlations with the shoot dry weight and chlorophyll content alterations in response to N-deficient condition. These results indicated that beside the chlorophyll content, the linolenic acid content and DBI may also contribute to N-deficiency adaptation, thus could be considered as efficient indicators for evaluation of different response in wheat seedlings under N-deficient condition.

**Conclusions:**

The alteration in fatty acid composition can potentially contribute to N-deficiency tolerance in plants, and the regulation of fatty acid compositions maybe an effective strategy for plants to adapt to N-deficient stress.

**Supplementary Information:**

The online version contains supplementary material available at 10.1186/s12870-020-02717-5.

## Background

Nitrogen (N) is a major limiting factor in crop growth, showing by decreased crop production after a reduction in the amount of N [1, 2]. Consequently, the modern agricultural production requires a large input of N fertilizer, which not only increases the agricultural production cost, but also enhances the potential risk to environmental pollution. The cultivation of high N-use-efficiency or N-deficiency tolerant genotypes is a primary approach to maintain crop production and reduce the application of N fertilizer [3, 4]. Therefore, understanding how plant response to N deficiency and the mechanism of N-deficiency tolerance are very important for sustainable development of modern crop production.

Plants quickly perceive and respond to the stress of N deficiency via a large number of physiological and metabolic events. Such as the degradation of proteins, decrease of the related enzyme activities, accumulation in carbohydrates, especially the starch, inducing oxidative stresses by generation of H_2_O_2_ and causing lipid peroxidation [5–7]. Among those events, the reduction of photosynthetic capacity is one of the most major N-deficiency-induced damages that inhibits plant growth and development [8]. Under N-deficient condition, not only the light-saturated photosynthetic rate and the quantum yield of photosynthesis were decreased [9, 10], but also the chlorophyll a and other pigment contents were all decreased after plants were suffered from N deficiency [11, 12]. Since photosynthesis is one of the key detrimental factors for plant biomass accumulation [13], and more than half of the total leaf N is allocated to the photosynthetic apparatus [14], it is important to understand how photosynthetic unit responses to N deficiency, especially in crops.

Photosynthetic membrane is the place where the light reaction occurred, and the regulation of membrane lipid and fatty acid composition is essential for plants growth and development [15, 16]. Several studies have been reported that plant can alter their membrane lipid compositions in response to various environmental stresses, such as drought, salt, heat and chilling stresses [17–22]. In maize, the alteration of galactolipids composition alleviated drought-caused leaf senescence and improved drought adaptation significantly [23]. In wheat, high temperature caused significant alterations in leaf lipid composition and unsaturation, and the heat-tolerant and susceptible genotypes exhibited different changes in their lipid composition and fatty acid unsaturation [24]. Thus, alteration in membrane lipid and fatty acid composition could be an effective strategy for plants to increase the tolerance to various environmental stresses, including N deficiency.

Previous studies showed that the compositions and contents of lipids were substantially affected by N deprivation in various species. In cynobacterium *Pseudanabaena sp*, galactolipids decreased with increasing of the phospholipid (i.e. phosphatidyl glycerol) under N deficiency [25]. When diatom was exposed to N deprivation, chloroplast membranes were extensively degraded, and the levels of galactolipids decreased dramatically [26]. In Arabidopsis, N deficiency led to a decrease in the chloroplast galactolipid composition, and the breakdown of galactolipids and chlorophyll was coordinated [27], suggesting the alteration in membrane lipid and fatty acid composition may play an important role in N deficiency responses. Recently, in soybean, a large reduction in galactolipid contents and great change in membrane lipid composition were found after exposed to N deficiency [28]. In wheat, the degradation of lipids and changes in lipid compositions were closely related with the N-deficiency induced leaf senescence [29, 30]. These studies indicate that N deficiency has a significant effect on plant membrane lipid contents and fatty acid compositions.

However, in higher plants, how the membrane fatty acid composition changes in response to N deficiency and what is the relationship with plant N-deficient tolerance are still unclear, especially in crops. In this study, seedlings of 24 modern commercial wheat cultivars were used to compare their different responses to N-deficient condition. Pearson’s correlation analysis and principal component analysis were applied to comprehensively evaluate their N-deficient tolerance. Our results showed that the changes in fatty acid composition as well as the chlorophyll content have a close relationship with the N-deficient tolerant responses in wheat seedlings. These results would contribute to a better understanding of the involvement of fatty acid remodeling in N-deficient tolerance, and provide useful information for future crop breeding in N-limited area.

## Results

### Responses of traits of growth and shoot N content to N-deficiency stress in wheat genotypes

**Fig. 1:**
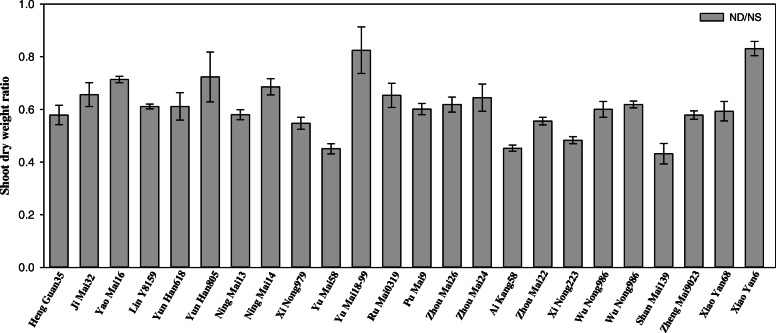
Changes in shoot dry weight in seedlings of 24 wheat cultivars after exposed to N deficiency. Data are represented with the ratio of N-deficient to N-sufficient treatments. NS represents the N-sufficient treatment (3.75 mM NH_4_NO_3_) and ND represents N-deficient treatment (0.375 mM NH_4_NO_3_). Data represent the mean ± SE (*n* = 3)

**Fig. 2:**
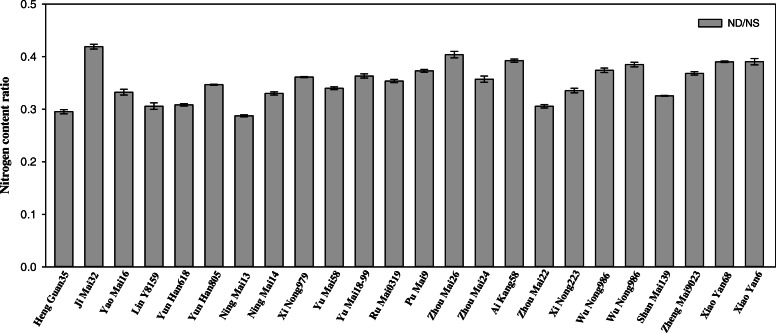
Changes in leaves nitrogen content ratio in seedlings of 24 wheat cultivars after exposed to N deficiency. Data are represented with the ratio of N-deficient to N-sufficient treatments. NS represents the N-sufficient treatment (3.75 mM NH_4_NO_3_) and ND represents N-deficient treatment (0.375 mM NH_4_NO_3_). Data represent the mean ± SE (*n* = 3)

When plants were exposed to N deficiency, their growth was significantly reduced (Table 1). The average values of shoot dry weight were 1.77 g and 1.06 g in N sufficient and N deficient conditions, respectively, in 24 wheat cultivars (Additional file 2). The shoot dry weight was reduced by N deficiency in all the cultivars (Fig. 1). The coefficient of variation (CV) of root dry weight and root length were higher than shoot dry weight, total dry weight and root/shoot ratio, indicating that N deficient stress had significant effects on the root growth in those 24 wheat cultivars. The same results were obtained in the shoot N content. N deficiency caused the decrease of shoot N content and different wheat cultivars have different responses (Fig. 2). There was no significant difference in shoot N content among 24 wheat cultivars after exposed to N deficiency.

**Table 1:** Alteration of traits of plant growth in 24 wheat cultivars

Genotype	Nitrogen treatment	Shoot dry weight (g/plant)	Root dry weight (g/plant)	Total dry weight (g/plant)	Root to shoot Ratio	Root length (cm)
Heng Guan 35	NS	1.69 ± 0.01	0.49 ± 0.03	2.18 ± 0.03	0.29 ± 0.02	29.7 ± 1.45
	ND	0.98 ± 0.06	0.73 ± 0.05	1.70 ± 0.12	0.74 ± 0.02	54.0 ± 0.58
Ji Mai 32	NS	2.09 ± 0.17	0.49 ± 0.04	2.58 ± 0.2	0.23 ± 0.00	39.0 ± 0.58
	ND	1.36 ± 0.01	0.94 ± 0.02	2.30 ± 0.04	0.70 ± 0.01	68.7 ± 0.67
Yao Mai 16	NS	1.67 ± 0.13	0.42 ± 0.04	2.09 ± 0.16	0.25 ± 0.00	46.7 ± 0.88
	ND	1.19 ± 0.07	0.87 ± 0.04	2.06 ± 0.11	0.74 ± 0.01	55.7 ± 0.88
Jin Mai 92	NS	2.01 ± 0.12	0.60 ± 0.06	2.61 ± 0.06	0.30 ± 0.05	29.7 ± 0.88
	ND	1.23 ± 0.09	0.73 ± 0.03	1.96 ± 0.10	0.60 ± 0.04	49.0 ± 0.58
Yun Han 618	NS	1.70 ± 0.06	0.46 ± 0.03	2.17 ± 0.04	0.27 ± 0.02	43.0 ± 0.58
	ND	1.04 ± 0.07	0.74 ± 0.04	1.78 ± 0.11	0.72 ± 0.01	67.7 ± 1.20
Yun Han 805	NS	1.64 ± 0.14	0.52 ± 0.04	2.16 ± 0.18	0.32 ± 0.01	37.0 ± 1.00
	ND	1.16 ± 0.06	0.67 ± 0.04	1.82 ± 0.10	0.58 ± 0.01	51.3 ± 3.53
Ning Mai 13	NS	2.32 ± 0.01	0.66 ± 0.01	2.97 ± 0.01	0.28 ± 0.00	40.0 ± 0.58
	ND	1.34 ± 0.04	0.75 ± 0.03	2.10 ± 0.05	0.56 ± 0.02	64.3 ± 1.45
Ning Mai 14	NS	2.06 ± 0.10	0.70 ± 0.01	2.76 ± 0.09	0.34 ± 0.02	34.3 ± 1.20
	ND	1.41 ± 0.03	0.78 ± 0.02	2.19 ± 0.05	0.55 ± 0.01	53.3 ± 0.88
Xi Nong 979	NS	1.70 ± 0.13	0.38 ± 0.01	2.08 ± 0.14	0.23 ± 0.01	21.0 ± 0.58
	ND	0.92 ± 0.03	0.51 ± 0.03	1.43 ± 0.05	0.55 ± 0.02	33.7 ± 0.88
Yu Mai 58	NS	1.86 ± 0.00	0.52 ± 0.05	2.38 ± 0.05	0.28 ± 0.02	35.3 ± 1.76
	ND	0.84 ± 0.04	0.55 ± 0.02	1.38 ± 0.05	0.65 ± 0.02	98.6 ± 1.21
Yu Mai 18–99	NS	1.21 ± 0.06	0.34 ± 0.02	1.55 ± 0.08	0.28 ± 0.01	47.0 ± 0.58
	ND	0.99 ± 0.07	0.81 ± 0.05	1.80 ± 0.13	0.82 ± 0.01	57.3 ± 0.67
Ru Mai 0319	NS	1.91 ± 0.09	0.52 ± 0.03	2.43 ± 0.12	0.27 ± 0.00	36.3 ± 0.33
	ND	1.24 ± 0.04	0.83 ± 0.03	2.07 ± 0.07	0.67 ± 0.01	55.3 ± 1.76
Pu Mai 9	NS	1.91 ± 0.04	0.57 ± 0.06	2.48 ± 0.04	0.30 ± 0.00	35.0 ± 1.73
	ND	1.15 ± 0.02	0.87 ± 0.03	2.02 ± 0.03	0.76 ± 0.04	60.0 ± 2.89
Zhou Mai 26	NS	1.87 ± 0.05	0.40 ± 0.02	2.27 ± 0.08	0.24 ± 0.01	35.0 ± 0.58
	ND	1.03 ± 0.02	0.75 ± 0.02	1.78 ± 0.04	0.73 ± 0.01	54.0 ± 1.15
Zhou Mai 24	NS	1.89 ± 0.06	0.51 ± 0.06	2.40 ± 0.06	0.27 ± 0.04	34.7 ± 0.88
	ND	1.21 ± 0.06	0.78 ± 0.03	1.99 ± 0.09	0.65 ± 0.02	53.3 ± 0.88
Ai Kang 58	NS	1.74 ± 0.04	0.47 ± 0.05	2.21 ± 0.08	0.27 ± 0.02	36.3 ± 0.88
	ND	0.79 ± 0.01	0.59 ± 0.01	1.38 ± 0.03	0.75 ± 0.01	81.7 ± 0.88
Zhou Mai 22	NS	1.87 ± 0.02	0.57 ± 0.03	2.44 ± 0.04	0.30 ± 0.02	36.0 ± 2.08
	ND	1.04 ± 0.02	0.57 ± 0.01	1.61 ± 0.03	0.55 ± 0.00	63.3 ± 3.84
Xi Nong 223	NS	2.09 ± 0.06	0.51 ± 0.01	2.59 ± 0.06	0.24 ± 0.00	25.0 ± 1.15
	ND	1.01 ± 0.01	0.65 ± 0.02	1.66 ± 0.03	0.65 ± 0.02	67.0 ± 0.58
Wu Nong 986	NS	1.68 ± 0.04	0.39 ± 0.03	2.07 ± 0.05	0.23 ± 0.01	34.3 ± 1.20
	ND	1.01 ± 0.03	0.59 ± 0.03	1.60 ± 0.06	0.59 ± 0.02	52.0 ± 1.15
Jun Mai 99–7	NS	1.65 ± 0.05	0.45 ± 0.01	2.10 ± 0.06	0.27 ± 0.01	34.7 ± 1.45
	ND	1.02 ± 0.02	0.76 ± 0.02	1.78 ± 0.04	0.74 ± 0.01	57.0 ± 0.58
Shan Mai 139	NS	1.67 ± 0.15	0.36 ± 0.04	2.03 ± 0.19	0.22 ± 0.00	34.7 ± 1.45
	ND	0.71 ± 0.02	0.42 ± 0.03	1.13 ± 0.04	0.59 ± 0.02	85.3 ± 0.88
Zheng Mai 9023	NS	1.60 ± 0.08	0.49 ± 0.04	2.09 ± 0.11	0.30 ± 0.01	30.0 ± 1.00
	ND	0.93 ± 0.07	0.66 ± 0.04	1.59 ± 0.11	0.71 ± 0.01	47.0 ± 1.15
Xiao Yan 68	NS	1.63 ± 0.10	0.45 ± 0.03	2.08 ± 0.13	0.28 ± 0.00	37.7 ± 1.45
	ND	0.96 ± 0.04	0.61 ± 0.02	1.57 ± 0.05	0.63 ± 0.01	62.0 ± 1.15
Xiao Yan 6	NS	1.14 ± 0.02	0.33 ± 0.02	1.47 ± 0.03	0.29 ± 0.02	45.0 ± 0.58
	ND	0.95 ± 0.02	0.60 ± 0.03	1.55 ± 0.05	0.63 ± 0.02	52.0 ± 1.15

NS and ND represented nitrogen sufficient and nitrogen deficient treatments, respectively. Data are means ± SE (*n* = 3)

### Responses of physiological parameters to N-deficiency stress in wheat genotypes

**Fig. 3:**
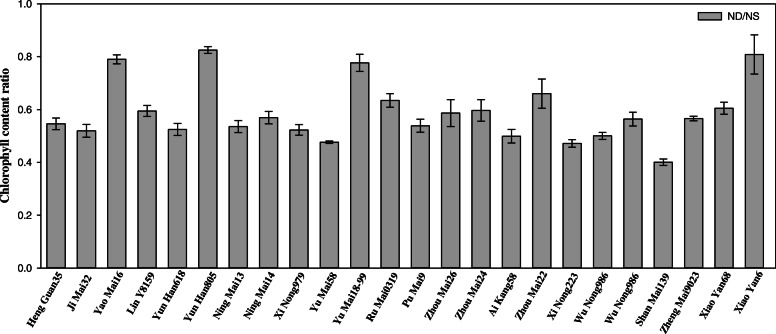
Changes in chlorophyll content ratio in seedlings of 24 wheat cultivars after exposed to N deficiency. Data are represented with the ratio of N-deficient to N-sufficient treatments. NS represents the N-sufficient treatment (3.75 mM NH_4_NO_3_) and ND represents N-deficient treatment (0.375 mM NH_4_NO_3_). Data represent the mean ± SE (*n* = 3)

In comparison with the N sufficient treatment, N deficiency decreased the chlorophyll content in all tested wheat cultivars, although the decrease extents were different (Table 2). In wheat cultivars Yao Mai 16, Yun Han 805, Yu Mai 18–99 and Xiao Yan 6, the chlorophyll contents were maintained higher compared to other cultivars, after N deficiency treatment (Table 2; Fig. 3). The electrical conductivity was increased in most wheat cultivars after N deficient treatment, but the increases were less pronounced in Yao Mai 16, Yun Han 805, Yu Mai 18–99 and Xiao Yan 6 cultivars. The levels of H_2_O_2_ and MDA in leaves were also measured under N-starved stress (Table 2). N deficiency had a pronounced effect on H_2_O_2_ accumulation, and this effect varied significantly in different genotypes. In some cultivars, including Yao Mai 16 and Xiao Yan 6, the H_2_O_2_ content only increased in a small amount, as compared with the same cultivar grown under control condition. While in other cultivars, including Yu Mai 58 and Xi Nong 223, the H_2_O_2_ content were increased by more than 100%, as compared with the N-sufficient condition. Similar change can be found in MDA content, which was obviously induced by N-deprived stress in all genotypes. We also investigated the changes in carbohydrate content under N deficiency (Table 3). It was shown that N deficiency increased the contents of soluble sugar, starch, total non-structural carbohydrates (TNC) as well as C/N ratio in all of the tested cultivars, but the changes of these parameters varied a lot in different genotypes.

**Table 2:** Alteration of physiological parameters in 24 wheat cultivars

Genotype	Nitrogen treatment	Chlorophyll content (mg/g DW)	Electrical conductivity	H_2_O_2_ content (μmol/g FW)	MDA content (μmol/g FW)
Heng Guan 35	NS	11.1 ± 0.05	0.14 ± 0.00	4.67 ± 0.19	6.38 ± 0.11
	ND	6.05 ± 0.27	0.20 ± 0.00	8.92 ± 0.12	10.5 ± 0.43
Ji Mai 32	NS	10.4 ± 0.26	0.13 ± 0.00	3.93 ± 0.10	4.28 ± 0.14
	ND	5.41 ± 0.19	0.20 ± 0.00	6.64 ± 0.41	8.21 ± 0.04
Yao Mai 16	NS	8.86 ± 0.22	0.14 ± 0.00	4.86 ± 0.68	5.80 ± 0.10
	ND	6.99 ± 0.04	0.17 ± 0.00	5.56 ± 0.12	7.55 ± 0.11
Jin Mai 92	NS	8.74 ± 0.13	0.21 ± 0.00	3.84 ± 0.86	3.98 ± 0.02
	ND	5.19 ± 0.12	0.26 ± 0.01	6.75 ± 0.21	8.01 ± 0.05
Yun Han 618	NS	8.70 ± 0.15	0.24 ± 0.00	3.00 ± 0.38	4.67 ± 0.01
	ND	4.56 ± 0.15	0.33 ± 0.00	5.78 ± 0.37	9.62 ± 0.49
Yun Han 805	NS	9.71 ± 0.04	0.27 ± 0.00	4.64 ± 0.63	5.95 ± 0.16
	ND	8.01 ± 0.10	0.24 ± 0.00	7.38 ± 0.50	9.83 ± 0.09
Ning Mai 13	NS	8.73 ± 0.11	0.20 ± 0.00	4.26 ± 0.25	5.18 ± 0.09
	ND	4.67 ± 0.15	0.51 ± 0.00	8.28 ± 0.35	10.6 ± 0.17
Ning Mai 14	NS	7.45 ± 0.24	0.27 ± 0.00	3.59 ± 0.37	3.01 ± 0.10
	ND	4.23 ± 0.15	0.39 ± 0.01	6.45 ± 0.18	7.28 ± 0.08
Xi Nong 979	NS	9.05 ± 0.07	0.18 ± 0.00	3.26 ± 0.22	4.53 ± 0.22
	ND	4.73 ± 0.15	0.48 ± 0.00	6.16 ± 0.28	9.49 ± 0.14
Yu Mai 58	NS	8.11 ± 0.03	0.16 ± 0.00	2.99 ± 0.06	4.57 ± 0.21
	ND	3.86 ± 0.03	0.35 ± 0.02	8.13 ± 0.47	10.6 ± 0.22
Yu Mai 18–99	NS	10.5 ± 0.45	0.15 ± 0.00	4.91 ± 0.76	6.84 ± 0.23
	ND	8.08 ± 0.03	0.22 ± 0.00	7.06 ± 0.27	9.63 ± 0.77
Ru Mai 0319	NS	8.72 ± 0.21	0.25 ± 0.00	3.10 ± 0.46	4.40 ± 0.51
	ND	5.53 ± 0.17	0.30 ± 0.00	5.55 ± 0.60	7.95 ± 0.08
Pu Mai 9	NS	8.14 ± 0.08	0.20 ± 0.00	3.56 ± 0.52	4.03 ± 0.16
	ND	4.39 ± 0.24	0.48 ± 0.00	6.90 ± 0.25	7.58 ± 0.10
Zhou Mai 26	NS	9.35 ± 0.32	0.16 ± 0.00	3.80 ± 0.53	4.39 ± 0.19
	ND	5.45 ± 0.31	0.34 ± 0.00	6.63 ± 0.40	9.32 ± 0.38
Zhou Mai 24	NS	8.86 ± 0.51	0.15 ± 0.00	3.34 ± 0.25	4.39 ± 0.23
	ND	5.25 ± 0.10	0.28 ± 0.00	6.89 ± 0.23	8.01 ± 0.09
Ai Kang 58	NS	7.80 ± 0.36	0.21 ± 0.00	3.13 ± 0.14	3.01 ± 0.04
	ND	3.98 ± 0.09	0.38 ± 0.00	6.47 ± 0.38	6.19 ± 0.16
Zhou Mai 22	NS	9.16 ± 0.16	0.21 ± 0.00	3.61 ± 0.55	4.15 ± 0.46
	ND	6.04 ± 0.42	0.31 ± 0.00	8.68 ± 0.35	10.0 ± 0.16
Xi Nong 223	NS	10.5 ± 0.25	0.18 ± 0.00	3.50 ± 0.30	3.76 ± 0.04
	ND	4.93 ± 0.05	0.31 ± 0.00	7.67 ± 0.22	8.45 ± 0.20
Wu Nong 986	NS	9.70 ± 0.12	0.13 ± 0.00	2.90 ± 0.18	4.41 ± 0.14
	ND	4.85 ± 0.07	0.25 ± 0.00	6.27 ± 0.09	7.14 ± 0.38
Jun Mai 99–7	NS	10.6 ± 0.15	0.27 ± 0.00	3.43 ± 0.86	4.70 ± 1.01
	ND	5.99 ± 0.19	0.43 ± 0.00	6.77 ± 0.15	8.40 ± 0.23
Shan Mai 139	NS	8.19 ± 0.13	0.15 ± 0.00	2.81 ± 0.06	3.56 ± 0.18
	ND	3.27 ± 0.13	0.26 ± 0.00	6.42 ± 0.29	8.86 ± 0.19
Zheng Mai 9023	NS	11.2 ± 0.04	0.12 ± 0.00	3.22 ± 0.21	3.18 ± 0.03
	ND	6.32 ± 0.10	0.19 ± 0.00	6.70 ± 0.44	7.27 ± 0.09
Xiao Yan 68	NS	12.4 ± 0.16	0.26 ± 0.00	3.30 ± 0.62	4.21 ± 0.14
	ND	7.50 ± 0.20	0.40 ± 0.00	6.53 ± 0.13	10.6 ± 0.26
Xiao Yan 6	NS	10.7 ± 0.15	0.24 ± 0.01	3.25 ± 0.33	5.63 ± 1.15
	ND	8.63 ± 0.68	0.27 ± 0.00	4.24 ± 0.07	7.73 ± 0.17

NS and ND represented nitrogen sufficient and nitrogen deficient treatments, respectively. Data are means ± SE (*n* = 3)

**Table 3:** Alteration of carbohydrates parameters in 24 wheat cultivars

Genotype	Nitrogen treatment	Soluble sugar content (mg/g DW)	Starch content (mg/g DW)	TNC content (mg/g DW)	Carbon/nitrogen ratio (%)
Heng Guan 35	NS	20.7 ± 0.38	38.9 ± 0.40	59.6 ± 0.8	0.99 ± 0.02
	ND	28.6 ± 0.12	55.1 ± 0.27	83.7 ± 0.2	4.71 ± 0.02
Ji Mai 32	NS	26.1 ± 0.12	35.3 ± 0.09	61.5 ± 0.2	1.21 ± 0.00
	ND	32.5 ± 0.80	49.8 ± 0.41	82.3 ± 0.3	3.87 ± 0.04
Yao Mai 16	NS	23.4 ± 0.57	38.2 ± 0.71	61.6 ± 1.2	1.03 ± 0.03
	ND	23.7 ± 0.22	42.8 ± 0.36	66.5 ± 0.6	3.34 ± 0.04
Jin Mai 92	NS	20.3 ± 0.39	36.0 ± 0.27	56.3 ± 0.4	0.91 ± 0.01
	ND	26.6 ± 0.66	46.1 ± 0.22	72.7 ± 0.5	3.86 ± 0.02
Yun Han 618	NS	24.6 ± 0.21	35.5 ± 0.38	60.1 ± 0.3	0.99 ± 0.01
	ND	32.9 ± 0.37	46.1 ± 0.32	79.0 ± 0.7	4.20 ± 0.06
Yun Han 805	NS	30.0 ± 0.44	45.4 ± 0.15	75.4 ± 0.4	1.22 ± 0.01
	ND	35.5 ± 0.85	51.3 ± 0.44	86.8 ± 0.3	4.05 ± 0.04
Ning Mai 13	NS	22.5 ± 0.26	42.8 ± 0.25	65.3 ± 0.5	1.00 ± 0.01
	ND	32.7 ± 0.69	62.8 ± 0.58	95.5 ± 0.6	5.06 ± 0.01
Ning Mai 14	NS	20.9 ± 0.26	35.9 ± 0.46	56.8 ± 0.4	0.91 ± 0.01
	ND	29.1 ± 0.42	46.7 ± 0.46	75.8 ± 0.7	3.67 ± 0.07
Xi Nong 979	NS	22.1 ± 0.17	41.2 ± 0.06	63.3 ± 0.2	1.03 ± 0.00
	ND	30.5 ± 0.72	56.0 ± 0.46	86.6 ± 0.4	3.89 ± 0.03
Yu Mai 58	NS	20.4 ± 0.15	39.3 ± 0.25	59.7 ± 0.4	1.08 ± 0.01
	ND	31.9 ± 0.64	62.6 ± 0.59	94.5 ± 0.1	5.05 ± 0.05
Yu Mai 18–99	NS	21.3 ± 0.40	39.6 ± 0.09	60.9 ± 0.5	1.03 ± 0.02
	ND	25.2 ± 0.85	47.0 ± 0.43	72.2 ± 0.7	3.37 ± 0.04
Ru Mai 0319	NS	22.4 ± 0.20	43.2 ± 0.20	65.7 ± 0.4	1.18 ± 0.01
	ND	30.8 ± 0.73	64.1 ± 0.67	95.0 ± 0.6	4.82 ± 0.03
Pu Mai 9	NS	23.3 ± 0.15	43.0 ± 0.27	66.3 ± 0.1	1.20 ± 0.01
	ND	29.3 ± 0.33	62.9 ± 0.09	92.2 ± 0.4	4.50 ± 0.01
Zhou Mai 26	NS	17.9 ± 0.09	40.9 ± 0.52	58.9 ± 0.4	1.05 ± 0.00
	ND	23.8 ± 0.75	52.5 ± 0.43	76.4 ± 0.4	3.37 ± 0.01
Zhou Mai 24	NS	22.6 ± 0.09	36.6 ± 0.09	59.2 ± 0.2	1.00 ± 0.01
	ND	28.4 ± 0.28	47.2 ± 0.35	75.6 ± 0.3	3.56 ± 0.02
Ai Kang 58	NS	19.7 ± 0.06	36.1 ± 0.36	55.8 ± 0.3	1.03 ± 0.00
	ND	33.1 ± 0.60	55.1 ± 0.40	88.2 ± 0.4	4.16 ± 0.01
Zhou Mai 22	NS	21.3 ± 0.12	37.6 ± 0.09	58.9 ± 0.0	1.06 ± 0.01
	ND	26.9 ± 0.09	60.5 ± 0.52	87.4 ± 0.5	5.16 ± 0.02
Xi Nong 223	NS	17.3 ± 0.62	32.8 ± 0.34	50.1 ± 0.5	0.90 ± 0.01
	ND	28.1 ± 0.61	63.6 ± 0.32	91.7 ± 0.0	4.92 ± 0.04
Wu Nong 986	NS	20.2 ± 0.49	36.3 ± 0.12	56.6 ± 0.4	1.07 ± 0.01
	ND	28.3 ± 0.72	57.2 ± 0.34	85.4 ± 0.4	4.32 ± 0.02
Jun Mai 99–7	NS	19.0 ± 0.34	35.5 ± 0.09	54.5 ± 0.4	0.97 ± 0.01
	ND	27.6 ± 0.69	58.8 ± 0.23	86.4 ± 0.2	4.01 ± 0.03
Shan Mai 139	NS	18.8 ± 0.22	36.6 ± 0.15	55.3 ± 0.4	1.04 ± 0.01
	ND	33.7 ± 0.19	52.6 ± 0.26	86.4 ± 0.4	4.99 ± 0.02
Zheng Mai 9023	NS	22.2 ± 0.38	42.8 ± 0.40	65.0 ± 0.7	1.18 ± 0.02
	ND	30.8 ± 0.72	61.3 ± 0.34	92.1 ± 0.0	4.53 ± 0.05
Xiao Yan 68	NS	23.3 ± 0.09	40.8 ± 0.36	64.1 ± 0.4	1.15 ± 0.01
	ND	29.4 ± 0.36	67.2 ± 0.23	96.6 ± 0.3	4.43 ± 0.02
Xiao Yan 6	NS	27.7 ± 0.38	50.4 ± 0.12	78.1 ± 0.4	1.43 ± 0.02
	ND	33.9 ± 0.82	60.8 ± 0.83	94.8 ± 0.8	4.45 ± 0.03

NS and ND represented nitrogen sufficient and nitrogen deficient treatments, respectively. Data are means ± SE (*n* = 3)

### Responses of fatty acid components to N-deficiency stress in wheat genotypes

We investigated the fatty acid components of leaf total lipids, including palmitic acid (16:0; number of carbon: number of double bonds in the acyl chain), hexadecylenic acid (16:1), hexadecadienoic acid (16:2), hexadecatrienoic acid (16:3), stearic acid (18:0), oleic acid (18:1), linoleic acid (18:2), and linolenic acid (18:3), as well as double bond index (DBI) under N-sufficient and deficient conditions (Table 4). It showed that 16:0 and 18:3 fatty acids are the primary lipid composition in wheat leaves. N deficiency increased the content of 16:0 fatty acid, while decreased the content of 18:3 fatty acid. The other fatty acids have relative low content, and they showed no significant changes among wheat genotypes after N deficient treatment. The double bond index (DBI) was reduced after N deficiency in most wheat cultivars.

**Table 4:**
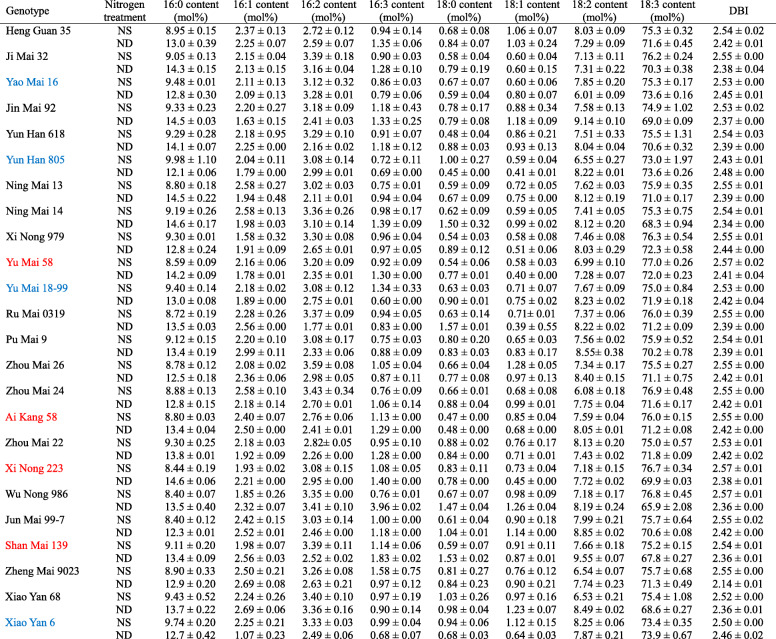
Alteration of fatty acid composition and DBI in 24 wheat cultivars

The words in blue color were grouped as low N-deficiency sensitive cultivars, the red color were grouped as high N-deficiency sensitive cultivars, while the black were moderated N-deficiency cultivars, based the principle component analysis in the present study. NS and ND represented nitrogen sufficient and nitrogen deficient treatments, respectively. Data are means ± SE (*n* = 3)

### Pearson’s correlation analysis to N-deficiency stress in wheat genotypes

Pearson’s correlation analysis was carried out to understand the relationship between fatty acid components and physiological parameters. As shown in Table 5, there were significant negative correlations between the shoot dry weight and carbohydrate levels, including sucrose (*r* = − 0.80), starch (*r* = − 0.66), and total non-structural carbohydrates (− 0.78), and also between the chlorophyll content and the carbohydrate levels (sucrose, *r* = − 0.78; starch, *r* = − 0.62; total non-structural carbohydrates, *r* = − 0.74). In contrast, carbohydrate contents showed strong positive correlations with root length, H_2_O_2_ and MDA contents. Looking at the correlation between fatty acid components and other physiological parameters, we found that 16:0 had a negative correlation with chlorophyll content (*r* = − 0.72), while had positive correlations with carbohydrate contents (*r* = 0.54, *r* = 0.66, *r* = 0.68 and *r* = 0.64 for contents of sucrose, starch, total non-structural carbohydrates and carbon to nitrogen ratio). Regarding to the 18:3, a significant positive correlation was found between 18:3 level and chlorophyll content (*r* = 0.78), in contrast, there were negative correlations between the levels of 18:3 and H_2_O_2_ (*r* = − 0.54), and starch content (*r* = − 0.56), respectively. Besides, the DBI was positively associated with chlorophyll content (*r* = 0.78), but negatively associated with starch content (*r* = − 0.57).

**Table 5:**
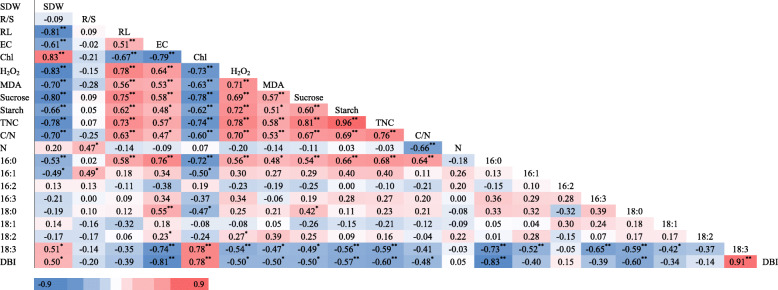
Correlation coefficient values (r) among multiple parameters in 24 wheat genotypes

All the data were based on the ratio of nitrogen deficient to nitrogen sufficient treatments. Scale: from brightest blue to brightest red represented negative and positive correlation, respectively, in the present study. ^**^ and ^*^ represent significance at 0.01 and 0.05 levels, respectively. SDW, shoot dry weight; R/S, root to shoot ratio; RL, root length; EC, electrical conductivity; Chl, chlorophyll content; H_2_O_2_, hydrogen peroxide content; MDA, malondialdehyde content; Sucrose, soluble sugar content; Starch, starch content; TNC, total non-structural carbohydrates; C/N, carbon to nitrogen ratio; N, leaf total nitrogen content; 16:0, palmitic acid; 16:1, hexadecylenic acid; 16:2, hexadecadienoic acid; 16:3, hexadecatrienoic acid; 18:0, stearic acid; 18:1, oleic acid; 18:2, linoleic acid; 18:3, linolenic acid; DBI, double bond index

### Comprehensive analysis of N-deficiency tolerance in wheat genotypes

**Fig. 4:**
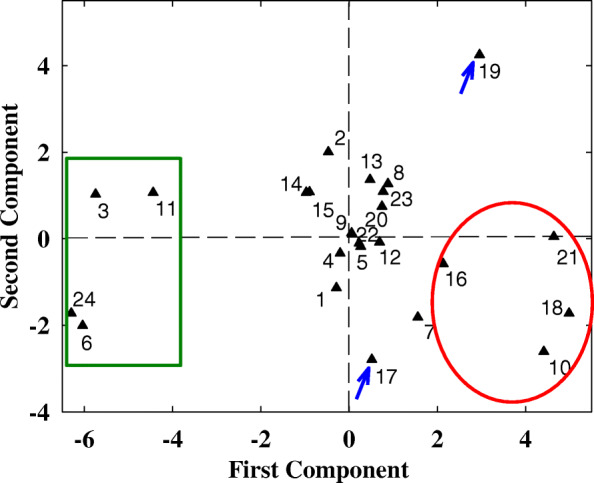
The scatter plot of 24 wheat cultivars basesed on the scores of the first two principle components. The values of shoot dry weight, root to shoot ratio, root length, electrical conductivity, chlorophyll content, hydrogen peroxide content, malondialdehyde content, soluble sugar content, starch content, total non-structural carbohydrates, carbon to nitrogen ratio, leaf total nitrogen content, palmitic acid, hexadecylenic acid, hexadecadienoic acid, hexadecatrienoic acid, stearic acid, oleic acid, linoleic acid, linolenic acid, and double bond index for the 24 cultivars were included in the principle component analysis. The wheat cultivars represented by the numbers are corresponded to those of Additional file 1. All the data are analyzed based on the ratio of N deficient to N sufficient treatments. The low N-deficiency sensitive cultivars are circled with green rectangle, the high N-deficiency sensitive cultivars are circled with red ellipse, the moderate cultivars, number 17 and 19 are shown with blue arrows

**Fig. 5:**
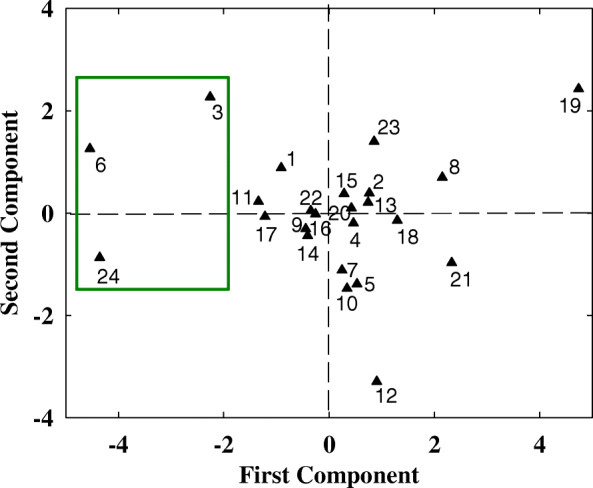
The scatter plot of 24 wheat cultivars basesed on the scores of the first two principle components. The values of palmitic acid, hexadecylenic acid, hexadecadienoic acid, hexadecatrienoic acid, stearic acid, oleic acid, linoleic acid, linolenic acid and double bond index for the 24 cultivars were included in the principle component analysis. The wheat cultivars represented by the numbers are corresponded to those of Additional file 1. All the data are analyzed based on the ratio of N deficient to N sufficient treatments. The low N-deficiency sensitive cultivars are circled with green rectangle

According to PCA results, the scores of the comprehensive indexes were obtained. In all traits, the first principle component featured with the N-deficiency tolerant genotypes, including Yao Mai 16, Yun Han 805, Yu Mai 18–99 and Xiao Yan 6, which were shown as number 3, 6, 11 and 24, and also the N deficiency sensitive genotypes, including Yu Mai 58, Ai Kang 58, Xi Nong 223 and Shan Mai 139, which were shown as number 10, 16, 18 and 21 in Fig. 4. The second principle component gave a high weighting to N-deficiency moderate genotypes, such as Zhou Mai22 and Wu Nong986 (number 17 and 19, respectively in Fig. 4). Furthermore, the PCA was conducted in fatty acid traits, the results showed that the first component had a higher eigenvalue in N-deficiency tolerant genotypes (Fig. 5), which was consistent with the results of PCA in all traits, indicating that fatty acid level could give a high proportion in evaluating the N-deficiency tolerance.

### Responses of PCA in different traits to N-deficiency stress in wheat genotypes

**Fig. 6:**
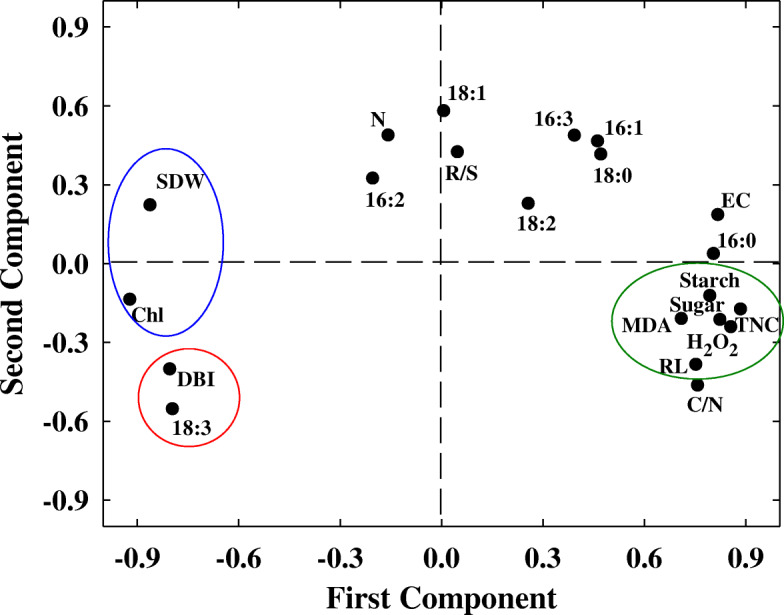
Principle component analysis (PCA) plot of 21 variables (the same variables used in Fig. 4) in 24 wheat cultivars. All the data are based on the ratio of N deficient to N sufficient treatments. SDW, shoot dry weight; R/S, root to shoot ratio; RL, root length; EC, electrical conductivity; Chl, chlorophyll content; H_2_O_2_, hydrogen peroxide content; MDA, malondialdehyde content; Sucrose, soluble sugar content; Starch, starch content; TNC, total non-structural carbohydrates; C/N, carbon to nitrogen ratio; N, leaf total nitrogen content; 16:0, palmitic acid; 16:1, hexadecylenic acid; 16:2, hexadecadienoic acid; 16:3, hexadecatrienoic acid; 18:0, stearic acid; 18:1, oleic acid; 18:2, linoleic acid; 18:3, linolenic acid; DBI, double bond index

Two principal components (principle component 1–2) were extracted with eigenvalues ≥1, and these components explained over 81% of the total variance in the dataset. As shown in Fig. 6, in all traits, the first principle component was strongly associated with total non-structural carbohydrates content, starch content, soluble sugar content, H_2_O_2_ and MDA contents, shoot dry weight and chlorophyll content, the second principle component gave a high proportion to 18:3 and DBI levels.

**Fig. 7:**
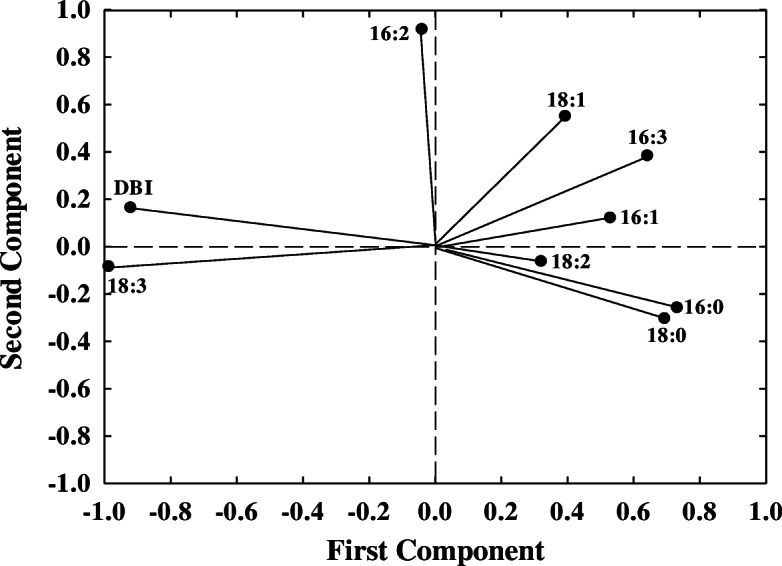
Principle component analysis (PCA) plot of 9 variables (the same variables used in Fig. 5) in 24 wheat cultivars. All the data are based on the ratio of N deficient to N sufficient treatments. 16:0, palmitic acid; 16:1, hexadecylenic acid; 16:2, hexadecadienoic acid; 16:3, hexadecatrienoic acid; 18:0, stearic acid; 18:1, oleic acid; 18:2, linoleic acid; 18:3, linolenic acid; DBI, double bond index

To further improve the accuracy of data analysis in fatty acid components, the fatty acid and DBI traits were subjected to PCA in 24 wheat cultivars. The results showed that the first principal components, featured with the largest contribution rate, were 18:3 and DBI (Fig. 7), which were consistent with all traits of PCA (Fig. 6).

## Discussion

Nitrogen is a major determinant for plant growth and lack of N nutrient dramatically decreases plant growth with other deleterious effects occur [31–33]. Previous studies have suggested that some physiological parameters, such as plant height, shoot dry weight and chlorophyll content, etc. could be useful indicators to evaluate the N-deficiency tolerance or sensitive in cereal, including spring wheat, barley and broomcorn [34–36]. These indices are recommended to quickly screening N-deficiency tolerant genotypes. Considering about different response to N deficiency during the different growth stages, although the most critical growth stages of wheat for N deficiency is the jointing to booting growth period [37, 38], the responses of wheat seedlings to N deficiency could also have a serious impact on the later stage of plant development [39, 40]. In the present study, the shoot biomass and chlorophyll content of seedlings were significantly changed in response to N deficiency in 24 wheat cultivars, and different cultivars showed different degrees of changes, suggesting that those two physiological indicators could also be the useful indicator in evaluation of wheat N deficiency response (Table 1; Table 2; Fig. 1; Fig. 3). Meanwhile, the root length, carbohydrates contents, damage in plasma membrane integrity, H_2_O_2_ accumulation and lipid peroxidation were all increased after exposed to N deficient stress, suggesting that the N-deficiency induced damages were occurred in those wheat seedlings, which were similar with previous studies [2, 7, 41–43].

Based on the data from 24 wheat cultivars and Pearson’s correlation analysis, we found that the changes of shoot biomass and chlorophyll content had close relationships with the levels of 16:0, 18:3 fatty acids, and DBI value (Table 5), indicating that except those widely used parameters (including the root length, sucrose and starch contents, and other damage indicators), the levels of 16:0, 18:3 fatty acids and DBI could also be considered as useful indicators for evaluation of plant N deficiency response. N deficiency could induce the production of long-chain saturated fatty acids [44]. The saturated and monounsaturated fatty acids accumulated when the N concentration was decreased [45]. It has been reported that N-deficient-tolerant soybean maintained higher chlorophyll content, as well as increased fatty acid metabolism [46]. Recently, in cultivated wheat, there were close relationships between the alteration in membrane lipid and fatty acid compositions and the plant N-deficiency response under N deficiency condition [31, 47]. The N deficiency caused the decrease of membrane lipid contents, especially the contents of 18:3 and 18:2 fatty acids, while the level of saturated fatty acid 16:0 was increased, lead to the significant decrease of DBI in wheat cultivars [29]. Moreover, it showed that the tolerant wheat cultivar had high chlorophyll content, high photosynthetic quantum yield of PSII and high photosynthetic rate compared to the sensitive one under N deficient condition, which could be ascribed to the maintenance of the membrane lipid content and the high DBI level [47].

To verify the above results, we conducted PCA of all physiological parameters in 24 wheat cultivars, three groups can be divided, the low N-deficiency sensitive group, including Yao Mai 16, Yun Han 805, Yu Mai 18–99 and Xiao Yan 6; the moderate N-deficiency sensitive group, including Heng Guan 35, Ji Mai 32, Jin Mai 92, Yun Han 618, Ning Mai 13, Ning Mai 14, Xi Nong 979, Ru Mai 0319, Pu Mai 9, Zhou Mai 26, Zhou Mai 24, Zhou Mai 22, Wu Nong 986, Jun Mai 99–7, Zheng Mai 9023 and Xiao Yan 68; and the high N-deficiency sensitive group, including Yu Mai 58, Ai Kang 58, Xi Nong 223 and Shan Mai 139 (Fig. 4 and Fig. 5). The low N-deficiency sensitive group exhibited significantly higher shoot dry weight and chlorophyll content than those of high N-deficiency sensitive group (Fig. 1 and Fig. 3), suggesting a less growth inhibition occurred in those cultivars under N deficient stress. In addition, the shoot dry weight, chlorophyll content, 18:3 fatty acid and DBI had maximum eigenvector load, which could be the identified indicators (Fig. 6 and Fig. 7). Meanwhile, the low N-deficiency sensitive cultivars had significantly lower level of 16:0 fatty acid and higher levels of 18:3 and DBI, while in high sensitive group, they showed opposite response (Table 4). Those results suggested that together with shoot dry weight and chlorophyll content, the levels of 16:0, 18:3 and DBI could be useful indicators for evaluation of plant N-deficiency responses.

Plant membrane lipids are primarily composed by 16-carbon and 18-carbon fatty acids, which are highly conserved in all plants [16]. In the present study, almost all wheat genotypes showed the significant increases in 16:0 fatty acid level and the decreases in 18:3 fatty acid and DBI levels after exposed to N deficiency (Table 4). Similar result has been reported in Arabidopsis that N deficiency had a great impact on membrane lipid turnover [27]. It has been shown that the changes in membrane lipid composition contributed to the maintenance of membrane structure and function, and lead to enhanced plant tolerance to various stresses [21, 23, 48, 49]. Overexpression of *OsMGD* improves salt tolerance in tobacco, and lipids play an important role in the plant salt stress response [21]. The regulation of lipid remodeling could be a promising strategy for improving drought adaptation in maize [23]. Moreover, it showed that the increase in saturated fatty acid level and the decrease in unsaturated fatty acid level were less in low N-deficiency sensitive cultivars, in other words, the low N-deficiency sensitive cultivars could maintain higher levels of fatty acids unsaturation than those high sensitive ones under N deficiency (Table 4), which could endow them a high capability to stabilize the membrane fluidity. Previous studies showed that the remodeling of fatty acid unsaturation under stress conditions was favorable to the stability and fluidity of the thylakoid membrane [50–52]. Also, increase in the level of unsaturated fatty acid was proved to contribute to a high plant drought tolerance [23, 53]. Therefore, the alteration of membrane lipid composition and fatty acid unsaturation could play a crucial role in enhancing N-deficiency tolerance in wheat plants.

The MGDG and DGDG are the two major galactolipids in leaves [54]. In plants, 16:3 is mainly found in MGDG while DGDG is mostly rich of 18:3 [55]. In the present study, when plants were exposed to N deficiency, the 16:3 content was decreased in low N-deficiency sensitive wheat cultivars (including Yao Mai 16, Yun Han 805, Yu Mai 18–99 and Xiao Yan 6), while it was significantly increased in high sensitive ones (Table 4), suggesting a decrease of MGDG in low N-deficiency sensitive cultivars but an increase in high sensitive ones. Similarly, 18:3 content showed unchanged or less decrease in low N-deficiency sensitive cultivars but a significant decreased in high sensitive cultivars under N deficiency. Taken together, those changes in 16:3 and 18:3 fatty acid contents could lead to a higher DGDG to MGDG ratio in low N-deficiency sensitive cultivars while a lower ratio in high sensitive ones. Since MGDG has a cone like shape and is easily to form a hexagonal-II (H_II_) phase, while DGDG has a more cylindrical shape and is a bilayer-forming lipid [56]. The relatively high DGDG to MGDG ratio in wheat could contribute to the stability of membrane system under N deficiency condition [29, 47]. It has been reported that the levels of DGDG was significantly higher in drought tolerant seedlings of *Cerastium fontanum* than that in drought sensitive *Lotus corniculatus* under drought stress condition [57]. Similar result has been shown in peanut and cowpea, in which the DGDG content was increased in tolerant cultivars, while it was decreased in sensitive genotypes under drought stress [17, 58]. Besides, the regulation of DGDG to MGDG ratio also contributed to enhanced stress tolerance in transgenic tobacco [21]. Therefore, the alteration in 16:0 and 18:3 fatty acid levels as well as DBI may be of beneficial to the stable of membrane structure, and lead to high tolerance to N deficiency.

In the current study, exposure of wheat plants to N-deficient stress resulted in significantly increases in the contents of H_2_O_2_ and MDA in high N-deficiency sensitive cultivars, as compared with the low N-deficiency sensitive ones (Table 2), suggesting that the high sensitive genotypes suffered more N-deficiency induced oxidative damages under N-deprived stress. Meanwhile, there was a negative correlation between MDA and 18:3 content (*r* = − 0.466), indicating that higher 18:3 fatty acid content may contribute to better maintenance of membrane structure and lead to less degradation in membrane lipid. Moreover, large evidences have shown that N deficiency could result in accumulation of carbohydrates (starch) in the leaves [5, 59, 60]. Excessive amounts of starch generally disrupt chloroplast structure and function, causing diminish in chlorophyll content, even have a feedback down-regulation of photosynthesis [8, 33, 61]. In the current study, we found either starch content or soluble sugar content were significantly increased under N-deprived stress, as well as total non-structure carbohydrates and C/N ratio. But the accumulation of soluble sugar and starch in low N-deficiency sensitive cultivars was less than that in high sensitive ones (Table 3), and they all showed clearly negative correlations with the levels of 18:3 fatty acid and DBI (Table 5). It has been shown that the less accumulatio of starch would benefit the maintenance of chloroplast ultrastructure, thus improved the plant growth in response to stresses [21, 23]. Therefore, it can be supposed that the less accumulation in carbohydrates could also contribute to less N-deficiency sensitive in the cultivars of Yao Mai 16, Yun Han 805, Yu Mai 18–99 and Xiao Yan 6.

In addition, due to the relatively large number of wheat cultivars used in this study, we could not investigate each component of membrane lipid, such as MGDG and DGDG, and this prevented us from comprehensively understanding the detailed responses of membrane lipids. But the strong correlation between fatty acid alteration and plant physiological responses under N deficiency could help us in understanding how lipids and fatty acids were involved in regulation of plant response to N deficiency. Moreover, it would be interesting to investigate the relation between year of cultivar released and lipid composition change, as a recent study showed that the newer wheat genotypes are more sensitive to low N compared with the older ones [62].

## Conclusions

In the present study, significant correlations were existed between the levels of shoot dry weight, chlorophyll content and 18:3 fatty acid as well as DBI in wheat seedlings after exposed to N deficiency. Through Pearson’s correlation analysis and principal component analysis, the responses of 24 wheat cultivars under N deficient stress was comprehensively evaluated, the results showed that the chlorophyll content, 18:3 fatty acid and DBI had close positive correlations with shoot dry weight, indicating that they could be considered as effective indicators in evaluation of plant N-deficiency responses. Our results also indicated that the alteration in fatty acid composition can potentially contribute to N-deficiency tolerance in plants, which could provide a novel strategy for N deficiency adaption in crops. Moreover, future studies using the contrasting genotypes like sensitive versus tolerant genotypes should be carried out to identify novel genetic components that are responsible for increasing 18:3 fatty acids and membrane unsaturation, thus improves the tolerance to N-deficient stress.

## Methods

### Plant materials, growth conditions and treatments

A total of 24 wheat cultivars were used in this study (Additional file 1). The seeds of different wheat cultivars were purchased from Yangling Sanqin Seed Industry Company Limited (Yangling. Shaanxi, China). Seeds were sterilized with 1% sodium hypochlorite for 10 min, followed by rinsing thoroughly with distilled water for five times. After sterilization, seeds were sown and germinated in vermiculite for 5 days. When the cotyledons expanded, the uniform seedlings from each cultivar were selected, after removing the seeds, the seedlings were transplanted into plastic containers (length × width × height: 40 × 30 × 15 cm) containing 5 L of half-strength Hoagland solution (pH 5.8) [63]. Each container included 20 seedlings of one genotype, and each genotype had 3 containers for each treatment. The whole nutrient solution consisted of: 3.75 mM NH_4_NO_3_, 0.5 mM KH_2_PO_4_, 1 mM MgSO_4_, 2.5 mM KCl, 2 mM CaCl_2_, 1 mM MgSO_4_, 1.5 × 10^− 6^ mM Fe-EDTA, 2.5 × 10^− 4^ mM MnCl_2_, 2.5 × 10^− 4^ mM ZnSO_4_, 1 × 10^− 4^ mM CuSO_4_, 1 × 10^− 4^ mM (NH_4_)_6_Mo_7_O_24_, 2.5 × 10^− 4^ mM H_3_BO_4_. Three days after transplanting, half of the seedlings were treated with N deficient (ND) Hoagland solution which contained 0.375 mM NH_4_NO_3_, and others were supplied with N sufficient (NS) Hoagland solution which contained 3.75 mM NH_4_NO_3_. The seedlings were sampled after 30 days of treatments, when 2–3 tillers had emerged. During the experiment process, the Hoagland solution was aerated and changed every 3 days. All the seedlings were grown in growth chambers at the same time that was set to a 14/10 h day/night cycle at a day/night temperature of 23/15 °C with 45 ~ 55% relative humidity, and the photosynthetic photon flux density was 500 μmol·m^− 2^·s^− 1^.

### Root length and biomass measurement

At the end of experiment, the longest root length of each plant was measured with a ruler. The shoot and root tissues of each cultivar from N deficient and sufficient treatments were sampled individually, then placed in an oven and dried at 80 °C for 3 days until a constant weight was reached. The shoot and root dry weight were measured, respectively. Three replications were included for each cultivar from each treatment and each replication included three plants from the same container.

### Determination of the contents of chlorophyll, leaf nitrogen and carbohydrate

Leaves (0.2 g) were extracted by 80% acetone on a shaker until the tissue was completely bleached. Then the extract was centrifuged at 5000 g for 5 min. After centrifugation, the supernatant was subjected to spectrophotometric measurements at 470 nm, 646 nm and 663 nm using a spectrophotometer (UV-2550 Shimadzu, Japan). The chlorophyll content was calculated according to the method of [64]. To measure leaf N content, leaf samples were dried and milled to powder. Then 0.1 g samples and 1.85 g K_2_SO_4_: CuSO_4_: Se = 100:10:1 catalyzer were added into the digestion tube, and added 5 mL sulfuric acid, then digested at 360 °C for 50 min until the solution was translucent, then cooled to room temperature. N content was measured by the standard macro-Kjeldahl procedure using a Kjeltec 2300 analyzer unit (Foss Tecator AB, Hoganan, Swedden).

The analysis of carbohydrate contents was according to [65–67]. Briefly, the 80% (v/v) ethanol were added into leaf powder (0.1 g) and kept at 80 °C for 30 min with constant stirring. After centrifugation, the supernatant was collected. The residue was further extracted by 80% ethanol at 80 °C for another two times. All supernatant was combined and the soluble sugar content was determined according to the method of [65]. The precipitate was used for starch extraction and the starch content was determined using the anthrone reagent according to [66]. The TNC was calculated as the total content of soluble sugars and starch. The carbon/nitrogen ratio was calculated as the ratio of TNC to N content according to [67].

### Determination of hydrogen peroxide (H_2_O_2_), lipid peroxidation and plasma membrane integrity

The H_2_O_2_ contents were measured spectrophotometrically according to [68] at 390 nm, with the extraction of leaf samples by 0.1% trichloroacetic acid at low temperature. The lipid peroxidation was estimated by measuring the malondialdehyde (MDA) contents in leaves, using the method of TBARS [69].

Plasma membrane integrity in wheat leaves was measured in terms of electrical conductivity (EC) according to [70]. The uniform fresh leaf discs were washed with deionized water to remove surface electrolytes, and then transferred into closed tubes containing 10 mL of deionized water. After incubation at 24 °C with 100 rpm in shaking for 24 h, the initial electrical conductivity (EC) (R1) of the solution was measured using EC meter (HORIBA Conductivity Meter B-173, Japan). Samples were then autoclaved at 120 °C for 20 min, and cooled down to room temperature, then the EC (R2) of the solution was recorded. The membrane stability index was defined as: MSI (%) = (R1/R2) × 100%.

### Fatty acid components analysis

Leave tissues (0.1 g) were extracted with 1 mL of 1 M methanolic HCl including 100 μL of pentadecanoic acid (15:0, pentadecanoic acid, Sigma) as internal standards. Then incubated at 80 °C for 30 min, cooling down for 5 min, and 1 mL of hexane and 1 mL 0.9% NaCl were added, then centrifuged at 1, 500 rpm for 3 min. Finally, the supernatant was collected and quantified by gas chromatography (GC-2010; Shimadzu, Japan) with flame ionization detector (FID) according to [47]. The fatty acid contents were quantified in contrast with the internal standards. The double bond index (DBI) was calculated as: DBI = [(16:1 mol% × 1) + (16:2 mol% × 2) + (16:3 mol% × 3) + (18:1 mol% × 1) + (18:2 mol% × 2) + (18:3 mol% × 3)]/100 [23].

### Statistical analysis

All data are represented as means ± SE of three replications. Data were analyzed using SPSS statistics software (Version 24.0 for Windows, SPSS, Chicago, USA). Pearson’s correlations were applied to evaluate the relationships between different physiological parameters and fatty acid compositions. All analyses of significance were conducted at the *P* < 0.05 level. In order to accurately assess physiological and fatty acid responses to N deficiency, principal component analysis (PCA) was performed using the ratio of N deficient to N sufficient treatments in 24 wheat cultivars.

## Supplementary Information

**Additional file 1: Table S1.** Genotype, year of release, breeding place and current commercial status of 24 wheat cultivars.

**Additional file 2: Table S2.**Variation of growth parameters in 24 wheat cultivars under nitrogen sufficient and nitrogen deficient treatments.

## Data Availability

The data that support the results are included within the article and its additional files. Other relevant materials are available from the corresponding authors on reasonable request.

## Abbreviations

N: Nitrogen
MGDG: Monogalactosyldiacylglycerol
DGDG: Digalactosyldiacylglycerol
TNC: Total non-structural carbohydrates
MDA: Malondialdehyde
H_2_O_2_: Hydrogen peroxide
EC: Electrical conductivity
PCA: Principal component analysis
16:0: Palmitic acid
16:1: Hexadecylenic acid
16:2: Hexadecadienoic acid
16:3: Hexadecatrienoic acid
18:0: Stearic acid
18:1: Oleic acid
18:2: Linoleic acid
18:3: Linolenic acid
DBI: Double bond index
