# Supplementary material for: Comprehensive evaluation of physiological traits under nitrogen stress and participation of linolenic acid in nitrogen-deficiency response in wheat seedlings
Source: BMC Plant Biol. 2020 Nov 3;20:501. doi: 10.1186/s12870-020-02717-5 (PMC7607636; doi:10.1186/s12870-020-02717-5)
Supplement: Supplementary file 1 — Additional file 1: Table S1. Genotype, year of release, breeding place and current commercial status of 24 wheat cultivars. [file 12870_2020_2717_MOESM1_ESM.docx]

Table S1. Genotype, year of release, breeding place and current commercial status of 24 wheat cultivars.

| Serial number | Genotype | Year of release | Breeding place | Current commerical status |
| --- | --- | --- | --- | --- |
| 1 | Heng Guan 35 | 2006 | Hebei | Yes |
| 2 | Ji Mai 32 | 2016 | Hebei | Yes |
| 3 | Yao Mai 16 | 2011 | Shanxi | Yes |
| 4 | Jin Mai 92 | 2013 | Shanxi | Yes |
| 5 | Yun Han 618 | 2010 | Shanxi | Yes |
| 6 | Yun Han 805 | 2011 | Shanxi | Yes |
| 7 | Ning Mai 13 | 2006 | Jiangsu | Yes |
| 8 | Ning Mai 14 | 2006 | Jiangsu | Yes |
| 9 | Xi Nong 979 | 2005 | Shaanxi | Yes |
| 10 | Yu Mai 58 | 2001 | Henan | Yes |
| 11 | Yu Mai 18-99 | 2003 | Henan | Yes |
| 12 | Ru Mai 0319 | 2009 | Henan | Yes |
| 13 | Pu Mai 9 | 2005 | Henan | Yes |
| 14 | Zhou Mai 26 | 2012 | Henan | Yes |
| 15 | Zhou Mai 24 | 2009 | Henan | Yes |
| 16 | Ai Kang 58 | 2005 | Henan | Yes |
| 17 | Zhou Mai 22 | 2007 | Henan | Yes |
| 18 | Xi Nong 223 | 2012 | Shaanxi | Yes |
| 19 | Wu Nong 986 | 2011 | Shaanxi | Yes |
| 20 | Jun Mai 99-7 | 2009 | Henan | Yes |
| 21 | Shan Mai 139 | 2011 | Shaanxi | Yes |
| 22 | Zheng Mai 9023 | 2003 | Henan & Shaanxi | Yes |
| 23 | Xiao Yan 68 | 2015 | Shaanxi | Yes |
| 24 | Xiao Yan 6 | 1981 | Shaanxi | Yes |
