# Supplementary material for: Comprehensive evaluation of physiological traits under nitrogen stress and participation of linolenic acid in nitrogen-deficiency response in wheat seedlings
Source: BMC Plant Biol. 2020 Nov 3;20:501. doi: 10.1186/s12870-020-02717-5 (PMC7607636; doi:10.1186/s12870-020-02717-5)
Supplement: Supplementary file 2 — Additional file 2: Table S2.Variation of growth parameters in 24 wheat cultivars under nitrogen sufficient and nitrogen deficient treatments. [file 12870_2020_2717_MOESM2_ESM.docx]

Table S2. Variation of growth parameters in 24 wheat cultivars under nitrogen sufficient and nitrogen deficient treatments.

| Traits | Nitrogen treatment | Variation range (Min-Max) | Mean | SD | CV (%) | Traits | Nitrogen treatment | Variation range (Min-Max) | Mean | SD | CV (%) |
| --- | --- | --- | --- | --- | --- | --- | --- | --- | --- | --- | --- |
| Shoot dry weight  (g/plant) | NS | 1.14-2.32 | 1.77 | 0.26 | 14.75 | TNC content  (mg/g DW) | NS | 50.10 -78.07 | 61.20 | 6.23 | 10.19 |
|  | ND | 0.71-1.41 | 1.06 | 0.18 | 16.82 |  | ND | 66.47-96.60 | 85.15 | 8.48 | 9.96 |
| Root dry weight  (g/plant) | NS | 0.33-0.70 | 0.48 | 0.09 | 19.47 | C/N ratio | NS | 0.90-1.43 | 1.07 | 0.12 | 11.39 |
|  | ND | 0.42-0.94 | 0.70 | 0.13 | 18.29 |  | ND | 3.34-5.16 | 4.26 | 0.57 | 13.47 |
| Total dry weight  (g/plant) | NS | 1.47-2.97 | 2.25 | 0.34 | 15.14 | 16:0  (mol%) | NS | 8.40-9.98 | 9.04 | 0.42 | 4.68 |
|  | ND | 1.13-2.30 | 1.76 | 0.29 | 16.36 |  | ND | 12.12 -14.64 | 13.44 | 0.77 | 5.75 |
| Root/shoot  ratio | NS | 0.21-0.34 | 0.27 | 0.03 | 11.42 | 16:1  (mol%) | NS | 1.58-2.58 | 2.21 | 0.24 | 10.86 |
|  | ND | 0.55-0.82 | 0.66 | 0.08 | 11.95 |  | ND | 1.07-2.99 | 2.17 | 0.41 | 18.99 |
| Root length  (cm) | NS | 21-47 | 35.72 | 6.14 | 17.19 | 16:2  (mol%) | NS | 2.72-3.59 | 3.19 | 0.22 | 6.93 |
|  | ND | 33.67-120.67 | 61.07 | 16.7 | 27.28 |  | ND | 1.77-3.41 | 2.66 | 0.42 | 15.89 |
| Shoot N content  (mg/g DW) | NS | 50.8-65.69 | 57.46 | 3.71 | 6.46 | 16:3  (mol%) | NS | 0.72-1.58 | 0.98 | 0.20 | 20.14 |
|  | ND | 16.94-22.66 | 20.13 | 1.58 | 7.86 |  | ND | 0.60-3.96 | 1.21 | 0.66 | 54.34 |
| Chlorophyll  (mg/g DW) | NS | 7.45-12.42 | 9.45 | 1.23 | 13.02 | 18:0  (mol%) | NS | 0.47-1.03 | 0.70 | 0.16 | 22.40 |
|  | ND | 3.27-8.63 | 5.58 | 1.42 | 25.42 |  | ND | 0.45-1.57 | 0.91 | 0.31 | 34.24 |
| Electrical conductivity | NS | 0.11-0.27 | 0.19 | 0.05 | 26.74 | 18:1  (mol%) | NS | 0.58-1.28 | 0.79 | 0.19 | 23.91 |
|  | ND | 0.17-0.51 | 0.31 | 0.10 | 31.11 |  | ND | 0.39-1.26 | 0.80 | 0.26 | 32.88 |
| H_2_O_2_ content  (μmol/g FW) | NS | 2.81-4.91 | 3.62 | 0.63 | 17.29 | 18:2  (mol%) | NS | 6.08-8.25 | 7.38 | 0.54 | 7.36 |
|  | ND | 4.24 -8.92 | 6.78 | 1.04 | 15.39 |  | ND | 6.01-9.55 | 8.02 | 0.70 | 8.75 |
| MDA content  (μmol/g FW) | NS | 3.01-6.84 | 4.54 | 1.00 | 22.01 | 18:3  (mol%) | NS | 72.97-77.03 | 75.57 | 0.96 | 1.28 |
|  | ND | 6.19-10.62 | 8.70 | 1.29 | 14.77 |  | ND | 65.92-73.87 | 70.80 | 1.89 | 2.67 |
| Soluble sugar (mg/g DW) | NS | 17.27-30.03 | 22.01 | 2.97 | 13.50 | DBI | NS | 2.43-2.57 | 2.54 | 2.81 | 1.11 |
|  | ND | 23.67-35.53 | 29.73 | 3.20 | 10.77 |  | ND | 2.34-2.48 | 2.40 | 3.26 | 1.36 |
| Starch content  (mg/g DW) | NS | 32.83-50.37 | 39.20 | 4.00 | 10.21 |  |  |  |  |  |  |
|  | ND | 42.80-67.20 | 55.43 | 7.10 | 12.81 |  |  |  |  |  |  |

CV, coefficient of variation; N, nitrogen; NS, nitrogen sufficiency; ND, nitrogen deficiency; H_2_O_2_, hydrogen peroxide content; MDA, malondialdehyde content; TNC, total non-structural carbohydrates; C/N ratio, carbon to nitrogen ratio; 16:0, palmitic acid; 16:1, hexadecylenic acid; 16:2, hexadecadienoic acid; 16:3, hexadecatrienoic acid; 18:0, stearic acid; 18:1, oleic acid; 18:2, linoleic acid; 18:3, linolenic acid; DBI, double bond index.
